# Supplementary material for: Dishevelled-1 regulates global transcriptomic changes and associates with ETS1 transcription factor
Source: Nat Commun. 2025 Jul 8;16:6288. doi: 10.1038/s41467-025-61551-1 (PMC12238645; doi:10.1038/s41467-025-61551-1)
Supplement: Supplementary file 1 — Supplementary Information [file 41467_2025_61551_MOESM1_ESM.pdf]

a

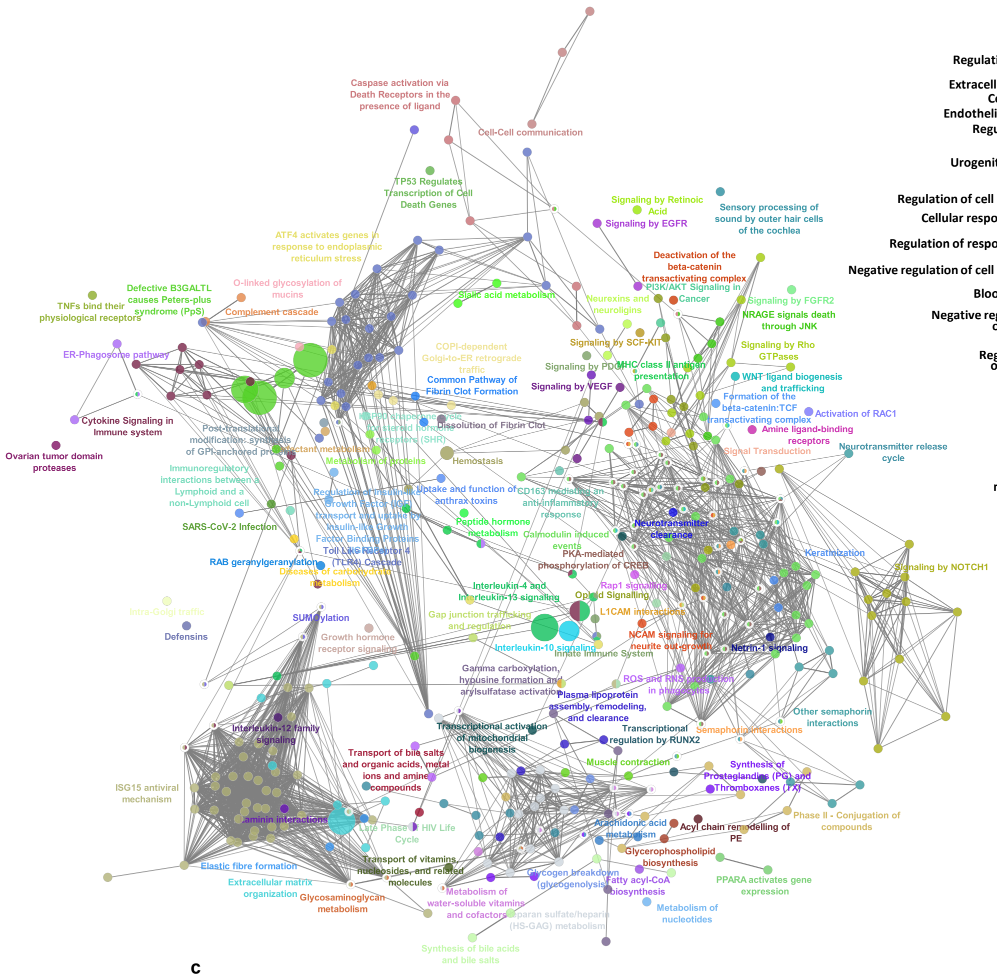

b

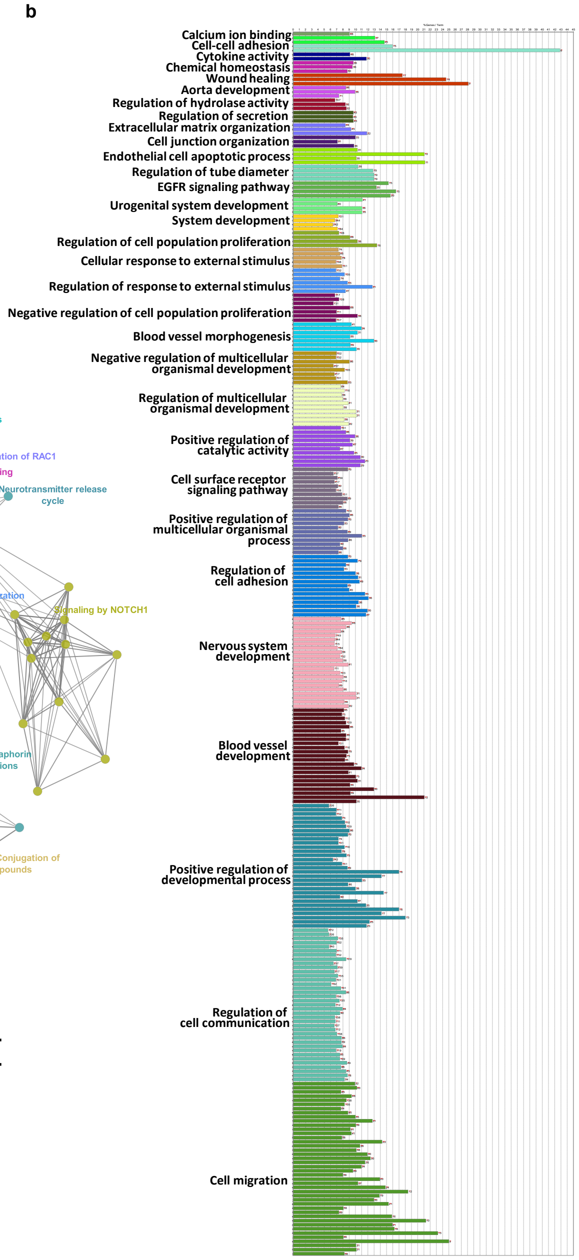

c

CLINVAR Disease Enriched Pathways	Term PValue
Age-related macular degeneration	0.011852
Arrhythmogenic right ventricular cardiomyopathy	0.001556
Beaded hair	3.89E-04
Brachydactyly type A2	0.015245
Branchiootorenal Spectrum Disorders	0.015245
Chronic obstructive pulmonary disease	0.005341
Familial hypercholesterolemia	0.016487
Familial thoracic aortic aneurysm and aortic dissection	0.031664
Genetic prion diseases	0.005341
Holoprosencephaly sequence	0.031664
Jakob-Creutzfeldt disease	0.005341
Melnick-Fraser syndrome	0.005341
Migraine	0.015245
Multiple sclerosis susceptibility	0.015245
Schizencephaly	0.015245
Schizophrenia	0.383704

Supplementary Figure 1. Overexpressed DVL1 alters numerous cancerous and non-cancerous pathways. (A) REACTOME pathway analysis of DEGs to identify enriched pathway changes ($n = 3$ biological replicates, log2FC > 2 or < −2, padj < 0.05, statistical analysis and p-value calculations were performed using ReactomePA). Each color represents a parental pathway, and its child-terms as associated nodes. Nodes that contain multiple colors have genes that are involved in several pathways. The size of the node represents the number of genes in each pathway. (B) Gene Ontology analysis of differentially expressed genes. Each color represents the parent GO term, and the bars represent the child GO term ($n = 3$ biological replicates, log2FC > 2 or < −2, padj < 0.05, statistical analysis and p-value calculations were performed using gProfiler). (C) ClinVar analysis on significant DEGs ($n = 3$ biological replicates, log2FC > 1 or < −1, padj < 0.05, statistical analysis and p-value calculations were performed using ClueGO). Source data are provided as the Source Data file.

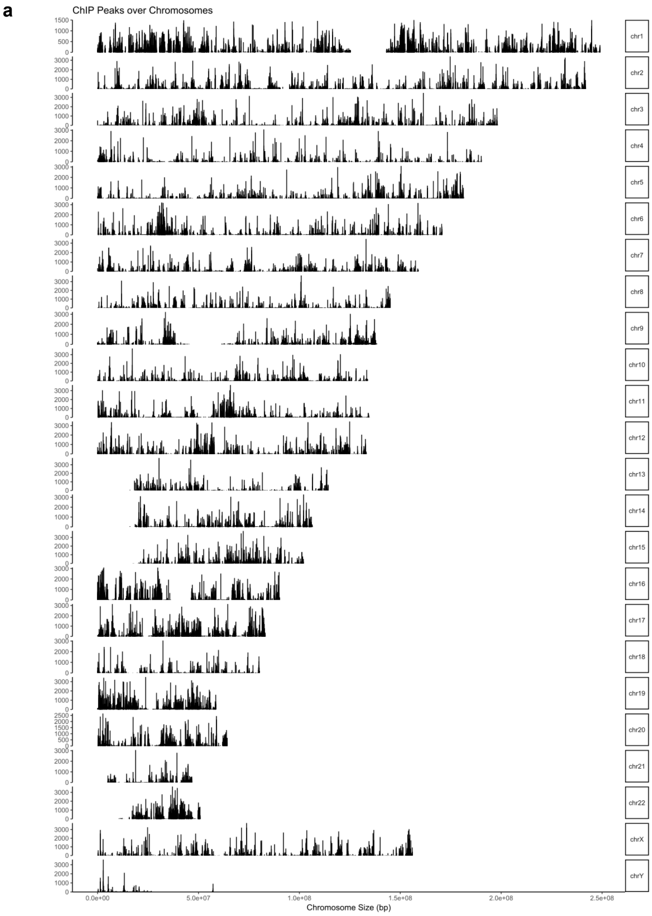

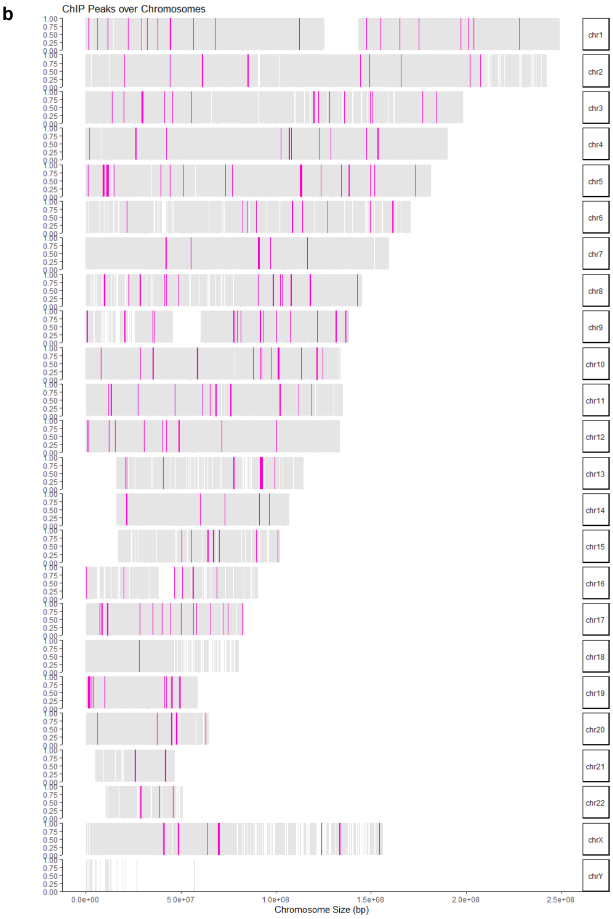

Supplementary Figure 2. Extensive DVL1 binding loci across MDA-MB-231 breast cancer cell line. DVL1 coverage plots depicting peak regions across chromosomes (*n* = 3 biological replicates, log2FC > 1 or < −1, padj < 0.05, peak identification and statistical analysis were performed using MACS2 and ChIPSeeker), featuring (A) peak heights at DVL1 binding loci, and (B) DVL1 coverage over WNT-related genes (pink) that interact with DVL1, while displaying other binding sites in grey. Source data are provided as the Source Data file.

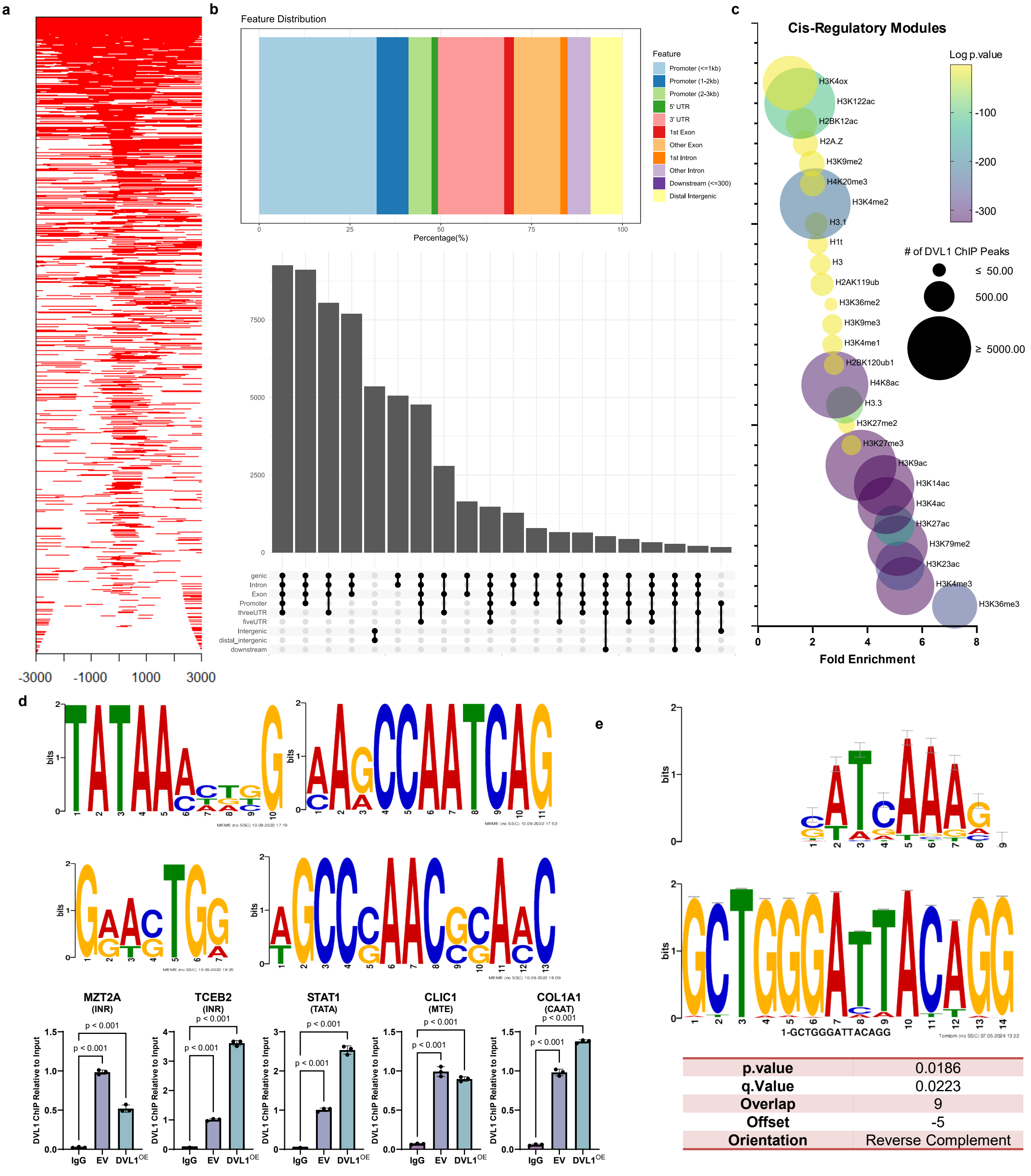

Supplemental Figure 3. Annotation of DVL1 binding loci in MDA-MB-231 breast cancer cell line. (A) Heatmap of DVL1 ChIP binding to transcription start site (TSS) region and proximal loci ($n = 3$ biological replicates, log2FC > 1 or < −1, padj < 0.05, peak identification and statistical analysis were performed using MACS2 and ChIPSeeker). (B) Annotation of the location of a given peak in terms of genomic features (*Top*), and to view the full genomic feature annotation with their overlap, we utilized an UpsetPlot (*Bottom*). The circles in each panel's matrix represent the different Venn diagram sections (gene features). Connected circles indicate a certain intersection of DVL1 peaks between features. The top bar graph in each panel summarizes the number of peaks for each unique or overlapping combination. (C) Cis-regulatory modules binding and expression target analysis to identify and quantify the enrichment of peaks from our DVL1 ChIP-seq data across different publicly available genomic datasets. (D) MEME motif analysis of ChIP sequences representing the different promoter regions (*Top:* CAAT, *Top Right*; TATA, *Top Left*; Inr, *Bottom* Left; MTE, *Bottom Right*), DVL1 ChIP qPCR of MDA-MB-231 cells with increased expression of DVL1 (WT^{OE}) compared to control cells (EV) in identified genes from ChIP-seq representing different promoter regions (*Bottom*) ($n = 3$ biological replicates (3 technical replicates each), mean ± SD, one-way, ANOVA followed by Dunnett's multiple comparison test was used to test significance). (E) STREME motif analysis of DVL1 ChIP sequences identified TCF4, a known member of the B-catenin/TCF transcriptional complex, binding loci. Source data are provided as the Source Data file.

a

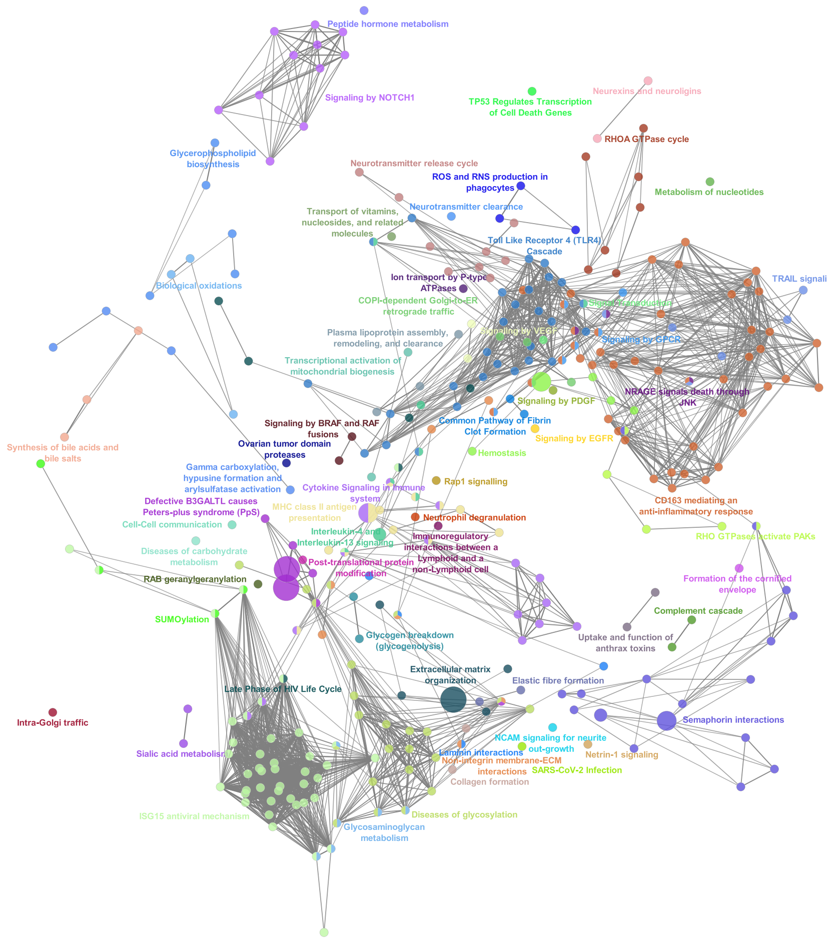
Peptide hormone metabolism
Signaling by NOTCH1
TP53 Regulates Transcription of Cell Death Genes
Neurexins and neuroligins
RHOA GTPase cycle
Glycerophospholipid biosynthesis
Neurotransmitter release cycle
ROS and RNS production in phagocytes
Metabolism of nucleotides
Transport of vitamins, nucleosides, and related molecules
Neurotransmitter clearance
Toll Like Receptor 4 (TLR4) Cascade
TRAIL signaling
Biological oxidations
Ion transport by P-type ATPases
COPI-dependent Golgi-to-ER retrograde traffic
Plasma lipoprotein assembly, remodeling, and clearance
Signaling by GPCR
Transcriptional activation of mitochondrial biogenesis
Signaling by PDGF
NRAGE signals death through JNK
Signaling by BRAF and RAF fusions
Common Pathway of Fibrin Clot Formation
Signaling by EGFR
Synthesis of bile acids and bile salts
Ovarian tumor domain proteases
Hemostasis
Gamma carboxylation, hypusine formation and arylsulfatase activation
Rap1 signalling
Cytokine Signaling in Immune system
CD163 mediating an anti-inflammatory response
Defective B3GALTL causes Peters-plus syndrome (PpS)
MHC class II antigen presentation
Neutrophil degranulation
Cell-Cell communication
Interleukin-4 and Interleukin-13 signaling
Immunoregulatory interactions between a Lymphoid and a non-Lymphoid cell
RHO GTPases activate PAKs
Diseases of carbohydrate metabolism
Post-translational protein modification
Formation of the cornified envelope
RAB geranylgeranylation
Complement cascade
SUMOylation
Glycogen breakdown (glycogenolysis)
Uptake and function of anthrax toxins
Extracellular matrix organization
Elastic fibre formation
Late Phase of HIV Life Cycle
Semaphorin interactions
Intra-Golgi traffic
NCAM signaling for neurite out-growth
Laminin interactions
Sialic acid metabolism
Non-integrin membrane-ECM interactions
Netrin-1 signaling
SARS-CoV-2 Infection
Collagen formation
ISG15 antiviral mechanism
Diseases of glycosylation
Glycosaminoglycan metabolism

b

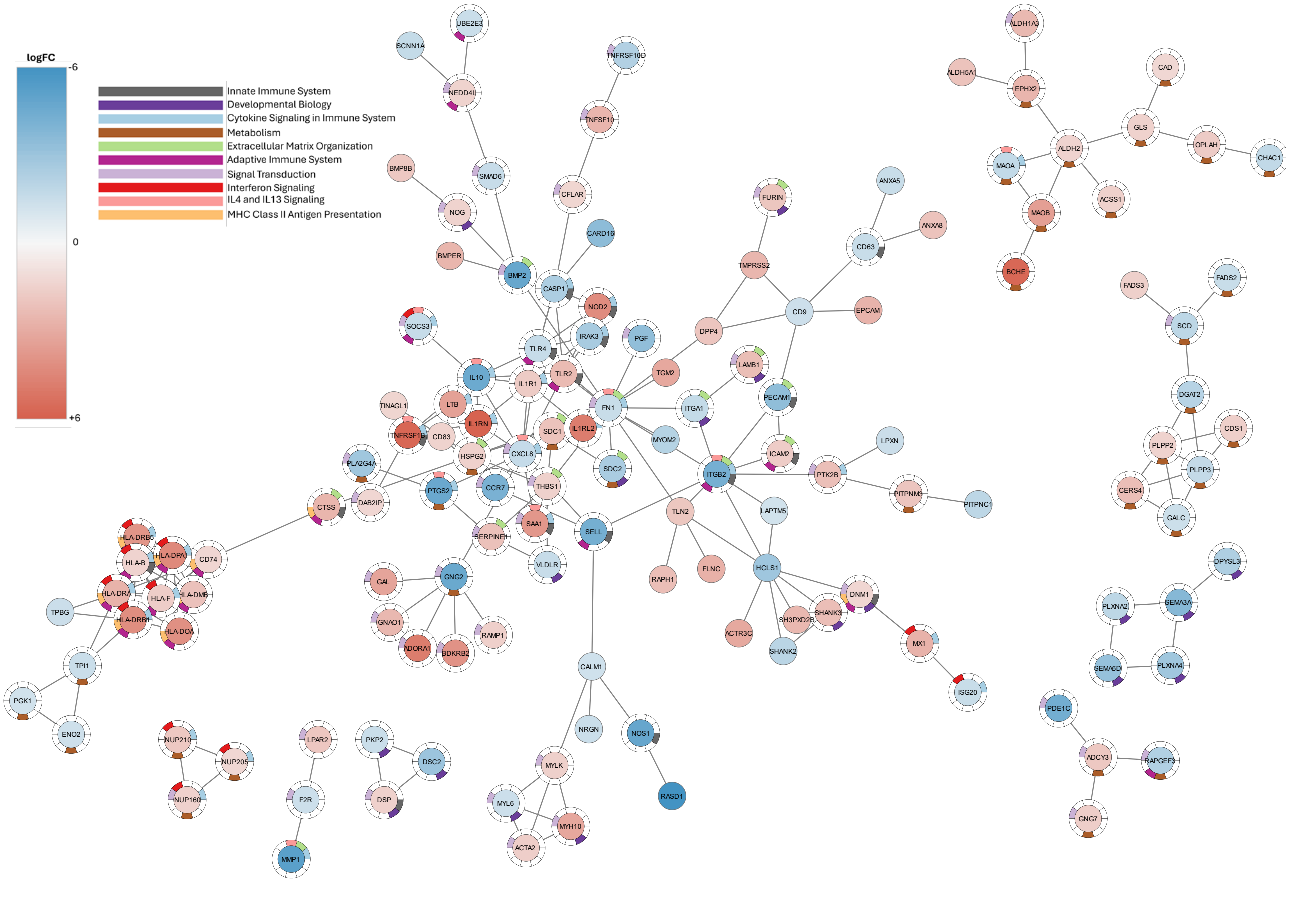
logFC
-6
0
+6
Innate Immune System
Developmental Biology
Cytokine Signaling in Immune System
Metabolism
Extracellular Matrix Organization
Adaptive Immune System
Signal Transduction
Interferon Signaling
IL4 and IL13 Signaling
MHC Class II Antigen Presentation

Supplementary Figure 4. Integrative Analysis of RNA-Seq and ChIP-Seq Identifies DVL1 Regulated Pathways. (A) REACTOME pathway analysis of overlapping RNA-Seq DEGS and DVL1 ChIP-Seq binding genes to identify enriched pathway changes ($n = 3$ biological replicates, log2FC > 1 or < −1, padj < 0.05, statistical analysis and *p*-value calculations were performed using ReactomePA). Each color represents a parental pathway, and its child-terms as associated nodes. Nodes that contain multiple colors have genes that are involved in several pathways. The size of the node represents the number of genes in each pathway. (B) Integrative STRING Enrichment ($n = 3$ biological replicates, log2FC > 1 or < −1, padj < 0.05, statistical analysis and *p*-value calculations were performed using STRINGdb, confidence score = 0.8)and REACTOME pathway analysis ($p<0.05$) of DVL1 RNA/ChIP-Seq DEGs and binding hits. Differentially expressed genes are represented by gene nodes visualized with continuous mapping by the logFC), and functional enrichment of the top 10 REACTOME pathways visualized by a split donut chart around each node. Source data are provided as the Source Data file.

a

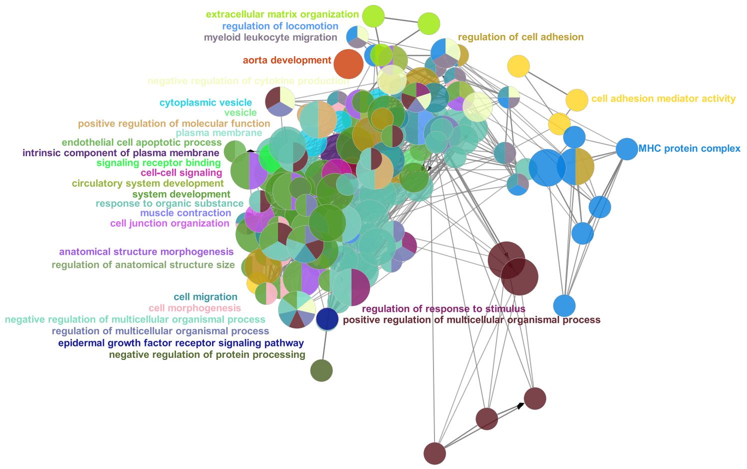

b

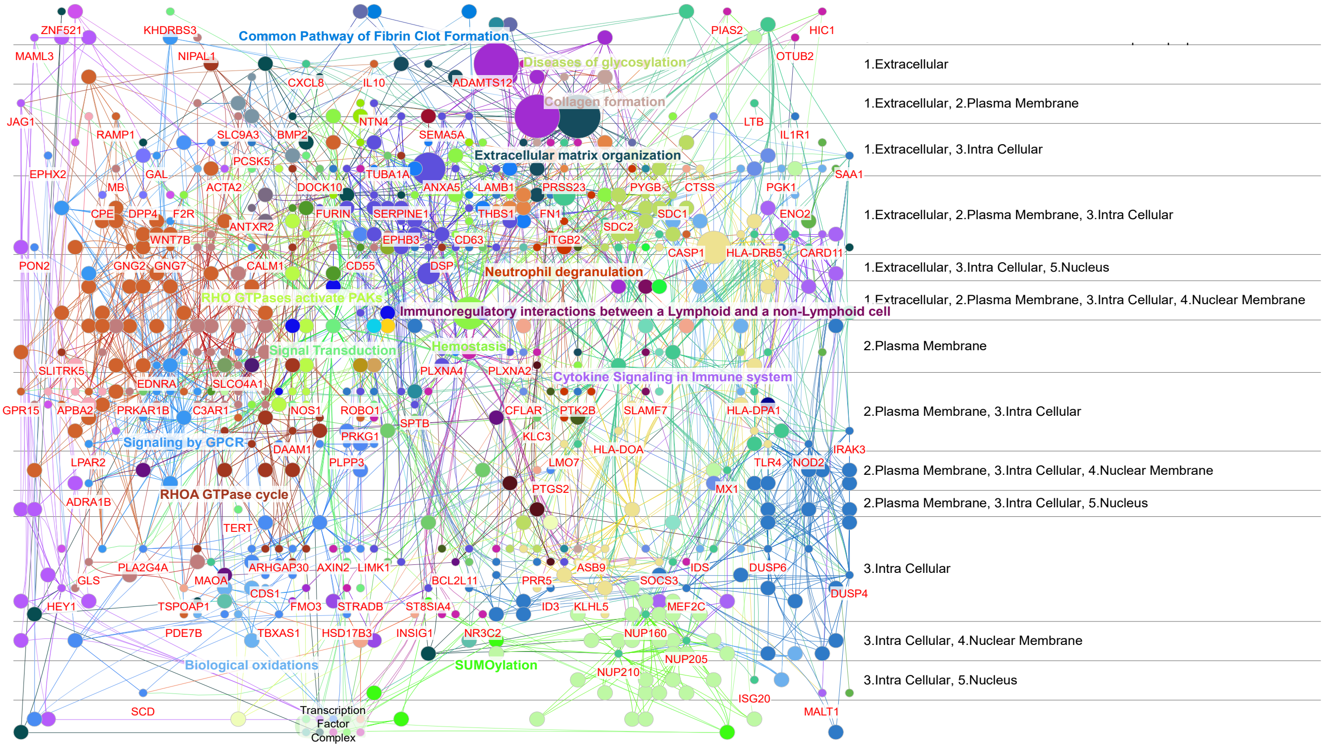

c

CLINVAR Disease Enriched Pathways	Term PValue
Acute myeloid leukemia	0.071326
Age-related macular degeneration	0.004174
Arrhythmogenic right ventricular cardiomyopathy	5.08E-04
Autism spectrum disorder	0.066729
Carcinoma of colon	0.236553
Chronic obstructive pulmonary disease	0.002963
Cutaneous melanoma	0.038414
Familial colorectal cancer	0.236553
Familial hypercholesterolemia	0.066729
Familial thoracic aortic aneurysm and aortic dissection	0.014449
Genetic prion diseases	0.002963
Hereditary cutaneous melanoma	0.038414
Jakob-Creutzfeldt disease	0.002963
Lissencephaly	0.038414
Malformation of cortical development	0.038414
Malignant melanoma of skin	0.038414
Migraine	0.008569
Multiple sclerosis susceptibility	0.008569
Myocardial infarction 1	0.15577
Schizophrenia	0.257207

Supplementary Figure 5. Integrative Analysis of DVL1 RNA-Seq and ChIP-Seq Identifies Altered Processes. (A) Gene ontology (GO) analysis of biological and molecular functions. Each color represents a parental pathway, and its child-terms as associated nodes. Nodes that contain multiple colors have genes that are involved in several pathways. The size of the node represents the number of genes in each pathway. changes ($n = 3$ biological replicates, log2FC > 2 or < −2, padj < 0.05, statistical analysis and *p*-value calculations were performed using Cytoscape). (B) Cell region-based rendering and layout annotation of overlapping DEGs and DVL1 ChIP-Seq hits. Genes are separated into layers according to their subcellular localization. Potential affected products of the pathway ($n = 3$ biological replicates, log2FC > 1 or < −1, padj < 0.05, statistical analysis and *p*-value calculations were performed using Cytoscape) are shown at the bottom of the view – transcription factor complex. (C) ClinVar analysis on significant DEGs ($n = 3$ biological replicates, log2FC > 1 or < −1, padj < 0.05, statistical analysis and *p*-value calculations were performed using ClueGO). Source data are provided as the Source Data file.

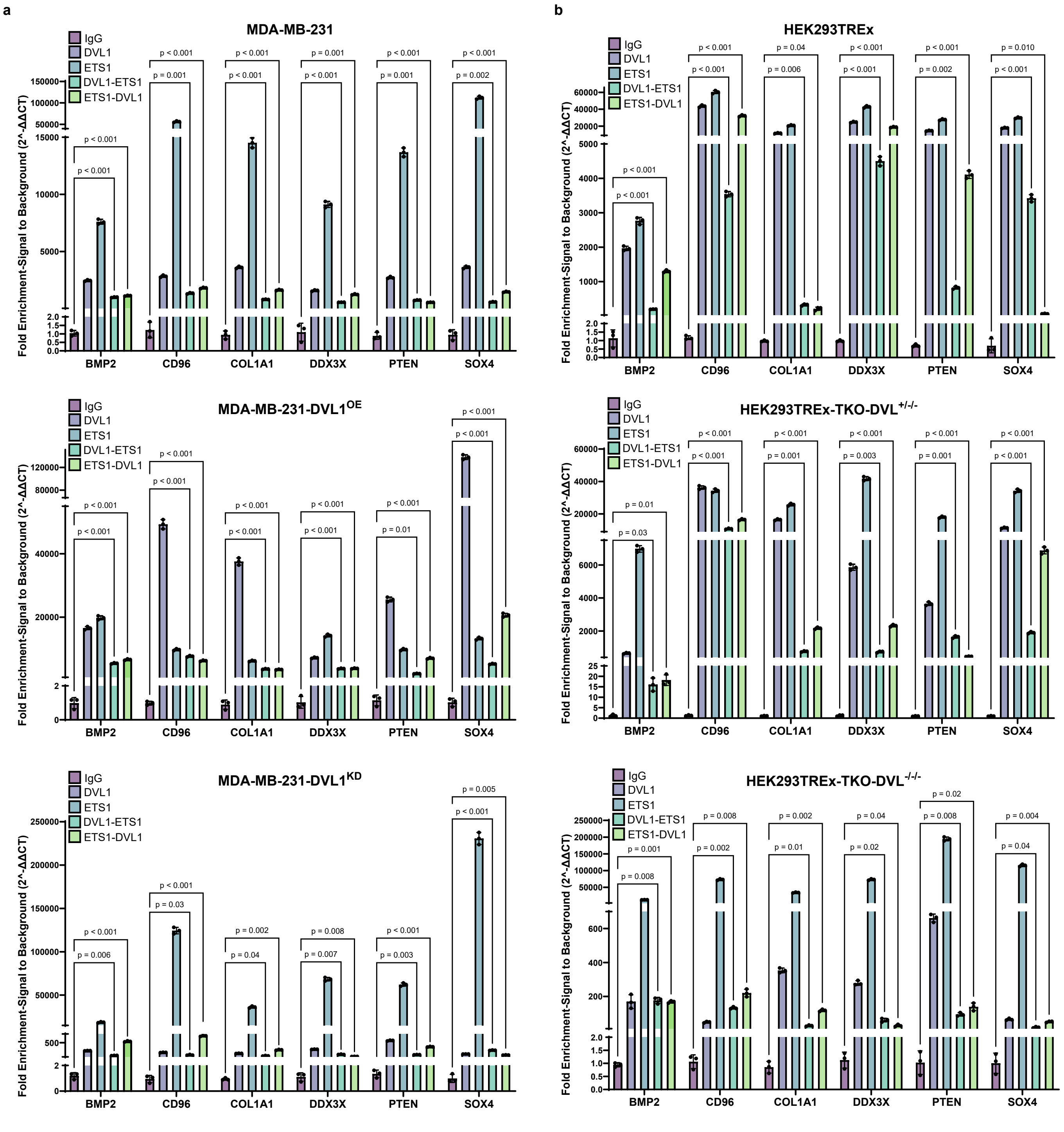

Supplementary Figure 6. DVL1 and ETS1 bind to the same genomic loci. (A) MDA-MB-231 and (B) HEK293TREx with increased (WT^{OE} and DVL1$^{+/-/-}$) or reduced (DVL1^{KD} and DVL$^{-/-/-}$) DVL1 expression. ChIP/re-ChIP-qPCR ($n = 3$ biological replicates (3 technical replicates each), mean ± SD, two-way, ANOVA followed by Dunnett's multiple comparison test was used to test significance) of endogenous DVL1, ETS1, in genes identified using integrative analysis of DVL1 ChIP/RNAseq and ETS1 regulated gene set list. Source data are provided as the Source Data file.

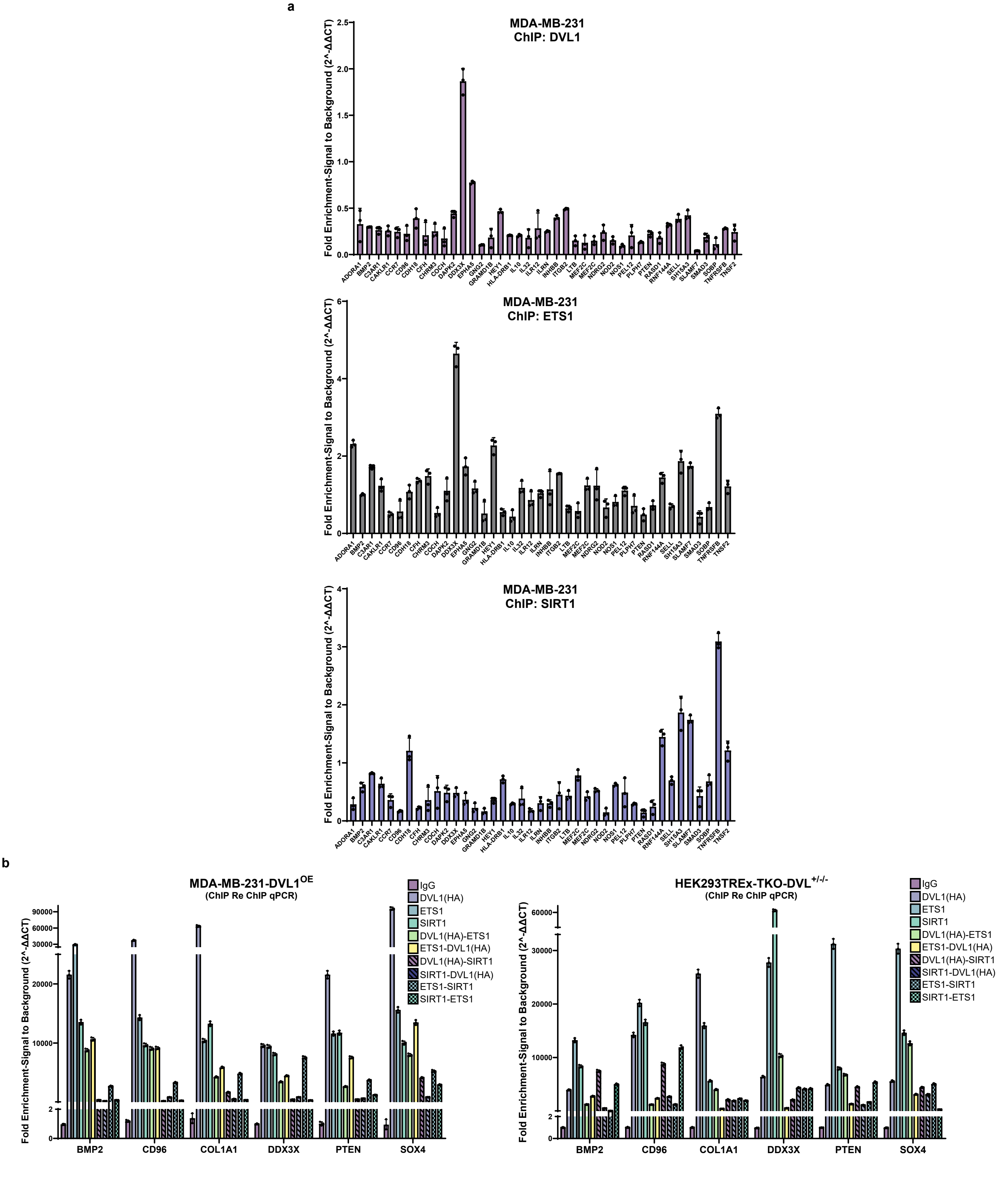

Supplemental Figure 7. SIRT1 binds to the same genomic loci as ETS1 and DVL1. (A) ChIP and (B) ChIP/re-ChIP qPCR ($n = 3$ biological replicates (3 technical replicates each), mean ± SD, two-way, ANOVA followed by Dunnett's multiple comparison test was used to test significance) show ETS1, DVL1 and SIRT1 binding to the same genomic loci on ETS1 regulated genes in MDA-MB-231 breast cancer cell line and HEK293TREx. Source data are provided as the Source Data file.

a

GSEA: Cancer Gene Neighborhoods/Cancer Modules				
	**logFC**	**AveExpr**	**t**	**Adj.P.Val**
**CDH18**	-7.2550	-2.9008	-11.1410	9.9375E-07
**APCS**	-6.5657	-3.0663	-8.5019	1.7704E-05
**SLAMF7**	-6.4941	2.4213	-15.2314	3.0428E-08
**LAIR2**	-6.4054	-1.0062	-7.2079	9.4318E-05
**CMKLR1**	-6.3193	1.4311	-21.4325	7.5269E-10
**SPANXC**	-6.1672	-2.6332	-7.1428	1.0171E-04
**PIWIL1**	-6.0872	-3.9757	-10.5139	1.8549E-06
**BMP3**	-6.0628	-2.3611	-7.8666	3.8784E-05
**KRT81**	-5.9115	6.5769	-31.1472	2.9221E-11
**VAT1L**	-5.8389	-2.9358	-6.8173	1.6308E-04
**RASD1**	-5.8309	4.3559	-24.8965	1.9747E-10
**TNS4**	-5.7216	4.9827	-25.8205	1.4603E-10
**PELI2**	-5.7066	1.2486	-9.1476	8.4591E-06
**CD96**	-5.6281	-2.0864	-8.5800	1.5973E-05
**CHRDL1**	-5.3845	-0.3432	-7.4579	6.6369E-05
**SHISA3**	-5.3403	0.4152	-14.4347	5.5749E-08
**ADAMTS1**	-5.2424	2.6739	-17.8389	5.4258E-09
**MMP1**	-5.0857	6.0838	-23.1499	3.6423E-10
**SPANXA1**	-4.9153	-3.8152	-5.7253	7.9636E-04
**SLC14A1**	-4.8968	-0.6568	-5.9506	5.6264E-04
**SLC1A7**	-4.8593	-1.1789	-6.3872	2.9336E-04
**SOBP**	-4.8504	-1.3215	-8.7426	1.3136E-05
**CHRM3**	-4.8128	-0.2309	-11.6063	6.3122E-07
**NOS1**	-4.6985	-4.0606	-5.9250	5.8146E-04
**BMP2**	-4.6613	1.6120	-13.7139	1.0098E-07
**RASD2**	4.4907	2.5235	12.2231	3.6058E-07
**TULP1**	4.5777	-1.7782	5.4987	1.1389E-03
**GJB3**	4.5786	1.5470	11.5240	6.7848E-07
**IL1RL2**	4.7127	-0.4174	5.0356	2.3636E-03
**ADORA1**	4.7261	-0.3913	4.5088	5.5349E-03
**RNASE1**	4.8256	-2.9726	5.2541	1.6659E-03
**MMP9**	4.8354	-0.4876	5.5554	1.0451E-03
**SERPINB7**	4.8846	-1.2645	5.9160	5.8851E-04
**DGCR5**	4.8870	0.3246	11.6975	5.7777E-07
**PAK6**	4.8887	0.9915	10.7856	1.3932E-06
**ARHGEF5**	4.9000	2.6928	12.1159	3.9080E-07
**WT1**	4.9536	0.2673	8.0737	2.9691E-05
**CD180**	4.9638	-4.1593	7.5463	5.8868E-05
**GCKR**	4.9640	-0.0730	6.2360	3.6628E-04
**SLC7A4**	5.0390	-2.9072	6.7801	1.6980E-04
**FNDC4**	5.4013	1.7324	10.0267	3.1044E-06
**GRAMD1B**	5.4998	0.9743	9.1097	8.8533E-06
**BCHE**	5.5367	-2.5485	5.2442	1.7070E-03
**TNFRSF1B**	5.5589	4.1418	21.3796	7.5269E-10
**ALOX5**	5.6977	1.8940	13.3446	1.3446E-07
**IL1RN**	5.7295	-2.5916	7.6230	5.2813E-05
**SPINK4**	5.9647	-0.9940	8.3008	2.2278E-05
**RNF144A**	6.2030	1.4442	12.6879	2.4053E-07
**CCBE1**	6.4728	2.7566	14.0869	7.5620E-08
**NGFR**	7.6650	3.4106	27.0124	9.8969E-11

b

GSEA: Hallmark Gene Set				
	**logFC**	**AveExpr**	**t**	**Adj.P.Val**
**SLAMF7**	-6.4941	2.4213	-15.2314	3.0428E-08
**CMKLR1**	-6.3193	1.4311	-21.4325	7.5269E-10
**CD96**	-5.6281	-2.0864	-8.5800	1.5973E-05
**ADAMTS1**	-5.2424	2.6739	-17.8389	5.4258E-09
**EPHA5**	-5.1532	0.0530	-5.4107	1.3219E-03
**MMP1**	-5.0857	6.0838	-23.1499	3.6423E-10
**PCDH7**	-4.8262	1.8992	-13.4787	1.2033E-07
**NOS1**	-4.6985	-4.0606	-5.9250	5.8146E-04
**BMP2**	-4.6613	1.6120	-13.7139	1.0098E-07
**PTGS2**	-4.5775	0.6303	-7.1396	1.0108E-04
**GNG2**	-4.5552	0.7436	-15.2340	3.0368E-08
**IL10**	-4.4945	-3.7237	-5.2871	1.5752E-03
**SERPINB2**	-4.3342	2.7663	-7.8401	3.9462E-05
**ALDH3A1**	-4.2511	-0.8882	-6.1763	3.9877E-04
**SELL**	-4.2051	-2.6488	-4.6870	4.0973E-03
**AGR2**	-4.1884	3.7723	-21.6028	7.1437E-10
**ITGB2**	-4.1743	3.2177	-22.3331	5.2573E-10
**CFH**	-4.1710	1.7530	-17.0601	8.6836E-09
**CCR7**	-4.0547	-1.1565	-6.0544	4.7907E-04
**HEY1**	-4.0284	0.3209	-7.9024	3.6775E-05
**HAS2**	-3.7008	3.2620	-15.7076	2.2383E-08
**INHBB**	-3.6498	3.7580	-16.3378	1.4297E-08
**NDP**	-3.6419	0.4442	-12.0925	3.9668E-07
**MEF2C**	-3.6305	1.8120	-8.9030	1.1132E-05
**PCSK9**	-3.6184	4.1081	-10.6864	1.5460E-06
**LTB**	3.3278	1.3472	5.5848	9.8811E-04
**SIRPA**	3.3318	1.1990	8.6473	1.4648E-05
**VNN1**	3.3324	1.0726	5.8620	6.3850E-04
**NDRG2**	3.3508	0.5884	5.9894	5.2472E-04
**C3AR1**	3.4662	-2.2091	3.7302	2.0787E-02
**EDN2**	3.6061	0.3657	9.3592	6.5285E-06
**P2RX6**	3.6912	0.5057	8.9254	1.0840E-05
**IL32**	3.6991	4.2997	18.9736	2.6546E-09
**TNFSF15**	3.7542	4.3059	16.7871	1.0322E-08
**VIPR1**	3.7605	3.6289	17.1248	8.5178E-09
**HLA-DOA**	3.9801	-1.0018	4.1028	1.0926E-02
**HLA-DRB1**	4.0682	3.5415	19.8850	1.7219E-09
**LRRC15**	4.0827	0.8027	9.0201	9.6436E-06
**NOD2**	4.1213	-2.2837	4.9623	2.6447E-03
**DAPK2**	4.1251	1.5065	8.7851	1.2612E-05
**COCH**	4.2738	-0.9748	3.5037	3.1589E-02
**CHST4**	4.3068	-1.1601	6.7935	1.6598E-04
**RGS4**	4.3888	2.7456	17.1758	8.3266E-09
**CXCR4**	4.4798	1.9557	9.5865	5.0952E-06
**GJB3**	4.5786	1.5470	11.5240	6.7848E-07
**IL1RL2**	4.7127	-0.4174	5.0356	2.3636E-03
**MMP9**	4.8354	-0.4876	5.5554	1.0451E-03
**GCKR**	4.9640	-0.0730	6.2360	3.6628E-04
**TNFRSF1B**	5.5589	4.1418	21.3796	7.5269E-10
**TFCP2L1**	6.5467	1.1259	10.8285	1.3562E-06

Supplementary Table 1. (EV vs WT^{OE}) GSEA: Cancer Gene Neighborhoods/Cancer Modules and Hallmark gene sets. Gene set enrichment analysis (GSEA) was performed to identify differentially expressed genes enriched in curated gene sets derived from large-scale cancer-related microarray datasets. These gene sets include signatures of commonly dysregulated pathways in cancer as well as hallmark gene sets representing distinct, well-characterized biological processes ($n = 3$ biological replicates, log2FC > 2 or < −2, padj < 0.05, statistical analysis and p-value calculations were performed using EdgeR). Source data are provided as the Source Data file.

Canonical						Non-Canonical
ADGRA2	DAB2IP	GLI3	MLLT3	SCEL	TRPM4	ANKRD6
AMER1	DAPK3	GNAQ	NDEL1	SCYL2	TTC21B	CCDC88C
AMFR	DDX3X	GPC3	NFKB1	SDC1	UBAC2	CSNK1D
ANKRD6	DIXDC1	GPC5	NKD2	SDHAF2	UBE2B	DAB2
APC	DKK2	GPRC5B	NKX2-5	SEMA5A	UBR5	DVL1
APC2	DKK3	GSK3A	NLE1	SFRP1	USP34	DVL2
APOE	DKK4	GSK3B	NOG	SFRP2	USP47	DVL3
APP	DLX5	GSKIP	NOTCH1	SFRP5	USP8	FZD1
ARNTL	DVL1	HDAC1	NPHP4	SHISA3	VCP	FZD2
ASPM	DVL2	HDAC2	NRARP	SHISA6	VPS35	FZD3
ATP6AP2	DVL3	HHEX	OTUD5	SIAH2	WLS	FZD5
AXIN1	EDA	IFT20	OTULIN	SLC9A3R1	WNK1	FZD6
AXIN2	EDNRA	ILK	PFDN5	SMAD3	WNK2	FZD7
BAMBI	EDNRB	INVS	PIN1	SNAI2	WNT10B	FZD8
BCL9	EGFR	ISL1	PLPP3	SOX13	WNT11	GPC3
BCL9L	EGR1	JADE1	PORCN	SOX4	WNT2	LRP6
BICC1	EMD	JRK	PPM1A	SOX9	WNT2B	MKS1
BTRC	EXT1	KANK1	PPM1B	SRC	WNT3A	MLLT3
CAPRIN2	FAM53B	KLF4	PPM1N	STK11	WNT4	ROR2
CAV1	FERMT1	KPNA1	PPP2R3A	STK3	WNT5A	RSPO1
CBY1	FGF10	KREMEN1	PRDM15	STK4	WNT5B	SFRP1
CCAR2	FGF2	LATS1	PRICKLE1	SULF2	WNT7A	SFRP2
CCDC88C	FGF9	LATS2	PRKN	TBL1XR1	WNT7B	SFRP5
CCNY	FGFR2	LEF1	PSEN1	TBX18	WNT8A	WNT11
CCNYL1	FGFR3	LGR4	PTEN	TCF7L1	WNT8B	WNT4
CDH1	FOXD1	LGR5	PTPRO	TCF7L2	WWTR1	WNT5A
CDH2	FOXO1	LIMD1	PTPRU	TGFB1	XIAP	WNT7A
CDK14	FOXO3	LMBR1L	PYGO1	THRA	YAP1	WNT7B
CHD8	FRMD8	LMX1A	PYGO2	TLE1	ZBED3	ZNRF3
COL1A1	FUZ	LRP4	RAB5A	TLE2	ZEB2	ABL1
CSNK1A1	FZD1	LRP5	RAPGEF1	TLE3	ZNRF3	ABL2
CSNK1D	FZD2	LRP6	RBMS3	TLE4		CELSR1
CSNK1G1	FZD3	LRRK1	RBPJ	TLE5		CELSR3
CSNK1G2	FZD5	LRRK2	RECK	TLE6		GRHL3
CSNK1G3	FZD6	LYPD6	RNF146	TMEM131L		MED12
CTDNEP1	FZD7	LZTS2	RNF220	TMEM64		MYOC
CTNNB1	FZD8	MAD2L2	ROR2	TMEM9		RAC1
CTNNBIP1	G3BP1	MCC	RSPO1	TNKS		RNF213
CTNND2	GATA3	MDK	RSPO2	TNKS2		RYK
CYLD	GID8	MESP1	RUVBL1	TNN		TIAM1
DAB2	GLI1	MKS1	RUVBL2	TPBG		VANGL2

Supplementary Table 2. WNT-associated binding loci identified in DVL1 ChIP-Seq. We analyzed the distribution of DVL1 binding peaks across genes associated with the Wnt signaling pathway. Our results revealed widespread DVL1 occupancy at Wnt-related loci, underscoring its prominent role in regulating this key signaling cascade ($n = 3$ biological replicates, log2FC > 1 or < -1, padj < 0.05, peak identification and statistical analysis were performed using MACS2). Source data are provided as the Source Data file.

ChIPseq-RNAseq: Top 25 Bidirectional				
	logFC	AveExpr	t	Adj.P.Val
**CDH18**	-7.25498	-2.90078	-11.141	9.94E-07
**SLAMF7**	-6.49412	2.42132	-15.2314	3.04E-08
**CMKLR1**	-6.31927	1.431148	-21.4325	7.53E-10
**RASD1**	-5.83092	4.35592	-24.8965	1.97E-10
**FAM155A**	-5.82213	-0.04692	-6.61137	0.000217
**LINC01559**	-5.7474	-1.62595	-7.37797	7.42E-05
**TNS4**	-5.72163	4.982738	-25.8205	1.46E-10
**PELI2**	-5.70658	1.248566	-9.14764	8.46E-06
**CD96**	-5.62808	-2.08638	-8.57995	1.60E-05
**SHISA3**	-5.3403	0.415236	-14.4347	5.57E-08
**ZC3H11B**	-5.28631	-1.16422	-6.39035	0.000293
**ADAMTS1**	-5.24238	2.673931	-17.8389	5.43E-09
**STK32A**	-5.2186	-1.96	-6.27006	0.000349
**EPHA5**	-5.15318	0.052975	-5.4107	0.001322
**MMP1**	-5.08572	6.083845	-23.1499	3.64E-10
**SPOCK3**	-4.93021	-0.46876	-8.44396	1.88E-05
**RAB37**	-4.91778	1.238798	-12.5507	2.69E-07
**SOBP**	-4.85045	-1.32153	-8.74261	1.31E-05
**PCDH7**	-4.82625	1.899218	-13.4787	1.20E-07
**CHRM3**	-4.81282	-0.23087	-11.6063	6.31E-07
**NOS1**	-4.69848	-4.06056	-5.92496	0.000581
**BMP2**	-4.66127	1.611953	-13.7139	1.01E-07
**ZNF804A**	-4.63538	0.905138	-6.06574	0.000472
**NTN4**	-4.58817	5.169959	-24.5606	2.14E-10
**PTGS2**	-4.57753	0.630277	-7.13961	0.000101
**PDE7B**	4.028016	1.761958	12.79036	2.23E-07
**HLA-DRB1**	4.068183	3.541469	19.88501	1.72E-09
**ADAMTS14**	4.070857	2.07936	11.73067	5.59E-07
**PIK3AP1**	4.108966	-0.25148	4.952742	0.00268
**NOD2**	4.121348	-2.28373	4.962326	0.002645
**DAPK2**	4.125053	1.506471	8.785105	1.26E-05
**NYNRIN**	4.171614	-2.2911	4.669423	0.004211
**SYNDIG1**	4.181783	-3.42816	4.829937	0.003269
**HLA-DPA1**	4.256929	3.516235	22.87044	3.93E-10
**COCH**	4.273832	-0.97476	3.503669	0.031589
**ACOXL**	4.310702	-0.05086	4.802888	0.003426
**EPHA10**	4.400268	0.074722	7.65903	5.04E-05
**ZNF727**	4.60606	-2.06024	4.434945	0.006314
**SLC28A3**	4.62074	-2.85472	5.116938	0.002076
**IL1RL2**	4.712668	-0.41739	5.03559	0.002364
**ADORA1**	4.7261	-0.3913	4.508788	0.005535
**MAGI2-AS3**	5.162858	0.310764	6.358461	0.000307
**FNDC4**	5.401295	1.732416	10.02667	3.10E-06
**GRAMD1B**	5.49976	0.974305	9.10968	8.85E-06
**BCHE**	5.53671	-2.54851	5.244156	0.001707
**TNFRSF1B**	5.558857	4.141812	21.37964	7.53E-10
**SEMA5A**	5.670925	-0.46188	5.772403	0.000747
**IL1RN**	5.729548	-2.59156	7.622986	5.28E-05
**LINC01088**	6.106142	-0.18755	9.846569	3.80E-06
**RNF144A**	6.203022	1.444203	12.68794	2.41E-07

Supplementary Table 3. Integrative Analysis of DVL1-ChIPseq and (EV vs WT^{OE}) RNAseq. Integration of RNA-seq and ChIP-seq data identified 633 genes that are differentially expressed and exhibit significant DVL1 binding enrichment. The top 25 logFC DEGs are shown. ($n = 3$ biological replicates, log2FC > 1 or < −1, padj < 0.05, statistical analysis and p-value calculations were performed using EdgeR). Source data are provided as the Source Data file.

LOLA: Transcription Factor					
**TF**	**maxRnk**	**meanRnk**	**rnkPV**	**rnkOR**	**rnkSup**
**GATA2**	1	1	1	1	1
**ETS1**	2	1.67	1	2	2
**TFAP2A**	10	5.67	1	6	10
**YY1**	14	6.33	1	14	4
**MZF1**	16	7.67	1	16	6
**ZEB1**	18	9.33	1	18	9
**FOXC1**	30	11.3	1	30	3
**ZNF354C**	26	11.7	1	26	8
**ARNT**	23	12.3	1	13	23
**NFIC**	29	12.3	1	29	7
**Klf4**	29	15.7	1	17	29
**SP1**	32	16	1	15	32
**HIF1A**	26	16.7	1	23	26
**MAFB**	35	18.3	1	35	19
**BRCA1**	43	18.7	1	43	12
**ZFX**	53	20.7	1	8	53
**HLTF**	60	22	1	60	5
**ELK1**	54	22.3	1	12	54
**SPIB**	53	22.3	1	53	13
**PAX2**	51	24.3	1	51	21
**SOX10**	59	24.7	1	59	14
**USF1**	39	24.7	1	34	39
**MYB**	41	25.3	1	41	34
**MYF**	57	26	1	20	57
**MYCN**	60	26.7	1	19	60
**GABPA**	77	27	1	3	77
**NFE2L1**	65	27	1	65	15
**NHLH1**	75	27.7	1	7	75
**ELK4**	76	28.7	1	9	76
**EBF1**	47	30.7	1	44	47
**MAX**	55	30.7	1	36	55
**NR4A2**	56	31.7	1	56	38
**EGR1**	82	41.3	37	5	82
**Nkx3-2**	68	44.7	38	68	28
**EN1**	55	45.7	36	55	46
**NFKB1**	80	47.3	41	21	80
**REL**	56	49.7	39	54	56
**NFATC2**	81	49.7	46	81	22
**CREB1**	62	50	40	48	62
**ZFP423**	81	50.3	43	27	81
**HAND1**	66	51	42	66	45
**E2F1**	88	51.3	44	22	88
**ELF5**	83	52.7	51	83	24
**FEV**	79	53	49	79	31
**MYC**	87	53.3	45	28	87
**MIZF**	104	53.7	53	4	104
**GATA3**	89	54.3	63	89	11
**INSM1**	89	55.7	47	31	89
**SPI1**	72	56.3	48	72	49

Supplementary Table 4. LOLA Transcription Factor analysis of DVL1 ChIP-Seq Locus overlap analysis (LOLA) was utilized to test overlap sets of genomic regions in our DVL1 ChIP-seq data and transcription factor profile matrices, to identify possible DVL1-transcription factor binding partners at the promoter regions. LOLA utilizes the Padj value, odds ratio (from a Fisher's exact test), and the raw number of overlapping regions to create an aggregate score and ranks each pairwise comparison for each of these statistics to calculate a combined rank for each factor. Source data are provided as the Source Data file.

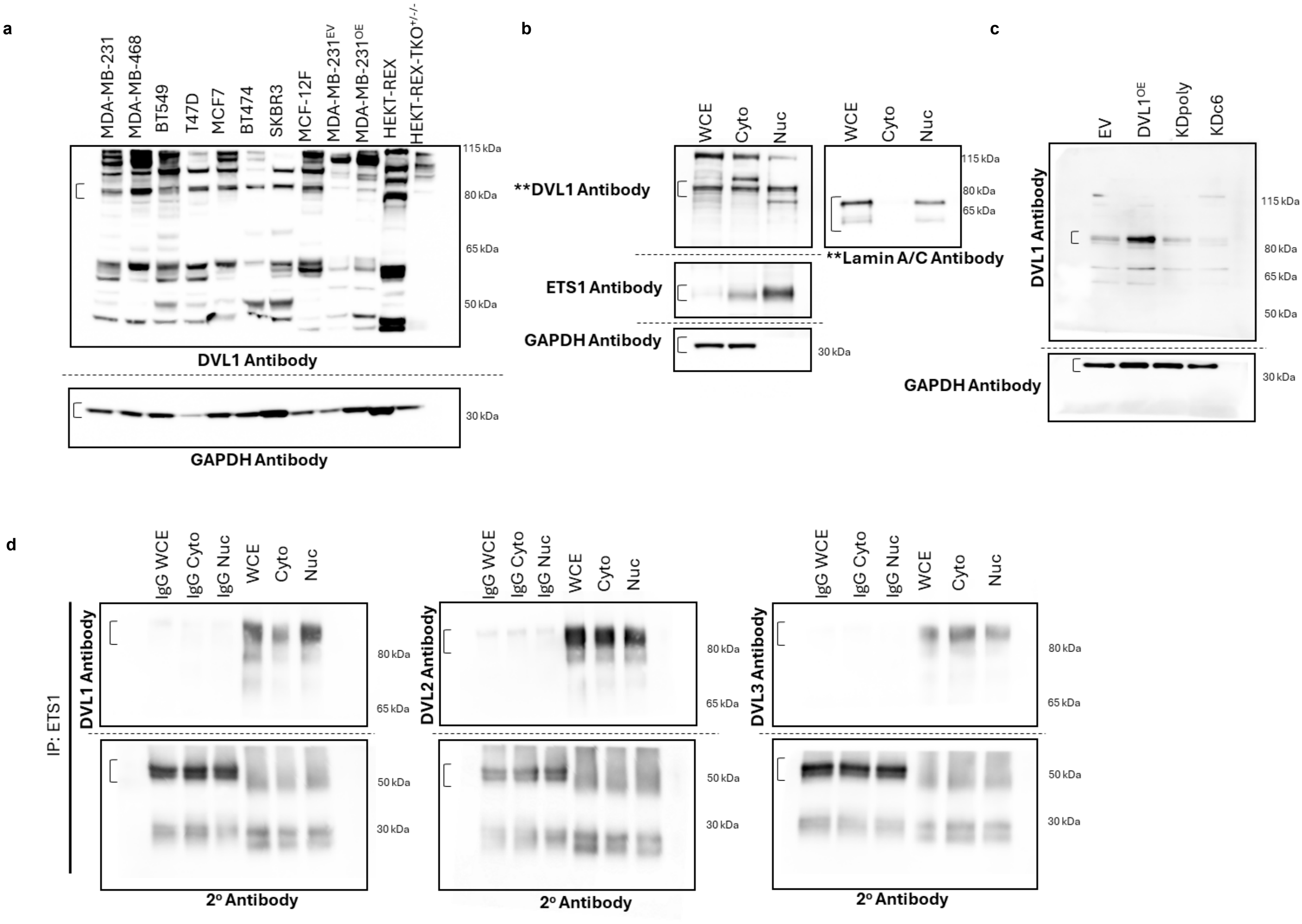

Supplementary Figure 8. Multiple antibodies were tested on the same gel/blot. Dotted lines indicate where the blots were cut horizontally between antibodies. **Blots that were stripped and re-probed are aligned side-by-side horizontally. (A) Western blot of DVL1 across multiple breast cancer cell lines. Representative blots shown (three biological replicates); GAPDH was the loading control. (B) Whole cell extract (WCE), cytosol (cyto) and nuclear (nuc) extract from the same biological sample were extracted and blotted for DVL1, ETS1 in MDA-MB-231. GAPDH was the loading control. (C) MDA-MB-231 empty vector (EV), overexpressed DVL1 (DVL1^{OE}), DVL1 knockdown of a polyclonal (KDpoly) and clonal population (KDc6) were blotted for DVL1 expression. GAPDH was the loading control. (D) Co-IP of ETS1 in MDA-MB-231 was performed and DVL1, DVL2, and DVL3 were blotted. 2º rabbit antibody was used to identify heavy and light chains for a loading control.
